# Potential of Insect Life Stages as Functional Ingredients for Improved Nutrition and Health

**DOI:** 10.3390/insects14020136

**Published:** 2023-01-28

**Authors:** Chrysantus M. Tanga, Hosea O. Mokaya, Wendie Kasiera, Sevgan Subramanian

**Affiliations:** International Centre of Insect Physiology and Ecology (*icipe*), Nairobi 00100, Kenya

**Keywords:** edible insects, *Gonimbrasia cocaulti*, *Bombyx mori*, *Samia Cynthia ricini*, micro- and macronutrients, entomophagy

## Abstract

**Simple Summary:**

Despite edible insects being possible future food sources, studies on the nutritional properties of some of the insects are still limited. *Gonimbrasia cocaulti* (GC), a saturniid caterpillar, is a delicacy in some communities in Africa, yet there is no information on its nutrition profile to provide insight on their contribution to rural household nutrition. This study provides in-depth knowledge on the nutritional composition of the edible larval stage of *G. cocaulti* and the pupal life stages of the domestic silkworm (*Bombyx mori*) and Eri silkworm (*Samia Cynthia ricini*). The results showed that the edible stage of GC had significantly high levels of linoleic acid, minerals (Ca, Fe, K), vitamins (B_6_, B_9_, B_12_, α-tocopherol) and crude fiber. The crude protein content of the larval and pupal stages of the various insects ranged between 50 and 62%. It is evident that the developmental life stage of GC and silkworms have adequate nutrients that could allow them to be utilized as ingredients in food fortification.

**Abstract:**

This study aimed to provide information on the nutrients of the edible larval stage of *Gonimbrasia cocaulti* (GC) for the first time, while exploring the potential nutrient content of the pupal life stages of the domestic silkworm (*Bombyx mori*; BM) and the Eri silkworm (*Samia Cynthia ricini*; SC). The three insects were analyzed for fatty acids, minerals, proximate composition and vitamins. Among the fatty acids, linoleic, a polyunsaturated fatty acid, was approximately threefold higher in GC than in the silkworms. The Ca, Fe and K contents were highest in GC. However, the Zn and Na contents were highest in BM, while Mg content was predominant in SC. The crude protein content of the various developmental life stages of the edible caterpillars and pupae ranged between 50 and 62%. Further, the fiber content of GC was substantially higher compared to the pupal stages of the two silkworm species. The vitamin (B_6_, B_9_, B_12_ and α-tocopherol) levels of the two insect life stages were considerably high. These insects are comparably rich in nutrients with potential suitability to be utilized in food fortification and thus ease pressure on the over-reliance on animal and plant-based sources, which are becoming unsustainable.

## 1. Introduction

With the world population expected to surpass nine billion by 2050, there is an urgent need to increase our current food production by almost 70% to feed this hungry population [1]. However, new agricultural land, which is necessary to support the production of resource-intensive animal and plant-based food products, is increasingly scarce [2]. Additionally, over 800 million people globally are reported to be undernourished, which further exacerbates the problems of food security especially in developing countries [3]. It would be unfair not to mention the negative effects of climate change on current food production systems, which further compound the challenges of feeding an expanding global population [4]. All of the aforementioned challenges point to the significance of identifying food sources and production practices that will not only leave a minimal environmental footprint and be nutritionally rich, but will also be able to adapt to the ongoing climate change-induced impacts on the environment.

Among the many alternatives, edible insects stand out as the most sustainable and innovative source of food and feed. Conventional nutritional sources (plants and animals) have economic shortfalls in their production and have more negative effects on the environment. For instance, livestock rearing contributes close to 36% of global agricultural greenhouse gas emissions [5], whereas plant cultivation leads to unsustainable conversion of forests into farming lands, extensive use of chemicals, exhaustion of water and loss of other vital biodiversity resources [6,7,8]. Therefore, sustainable yet nutritionally promising alternative sources such as edible insects should be encouraged as they require less land and water and have less negative impacts on the environment. For instance, one gram of beef protein requires over eight times more land and almost five times more water than the rearing of mealworms [9].

Globally, over 1900 insect species form part of human diets, with others being used as animal feed, an indication that entomophagy is widely practiced [7]. From a nutritional perspective, edible insects are not inferior to conventional sources but rather rival or are even better than them in terms of other nutrients [10]. For instance, edible caterpillars have higher protein content compared to chicken meat (28 and 21 g/100 g on a fresh matter basis, respectively) [11]. Furthermore, the digestibility of insect proteins is as good as that of the milk protein casein and soybean proteins [12]. Equally, insects contribute a wide spectrum of vitamins, fatty acids and minerals, all of which are essential in combating various deficiencies and preventing metabolic diseases [13,14,15]. Edible insects may also be referred to as ‘nutraceutical foods’, as they are not only nutrient dense but also contain natural substances (antioxidants) that provide functional benefits to the body [16,17,18].

*Gonimbrasia cocaulti*, a saturniid caterpillar, is a delicacy in some communities in Kenya and in other parts of Africa [19], yet there is no documentation on the nutritional composition of the edible larval stage. Though *G. cocaulti* was first described in Africa in 1992 [20], previous studies have largely focused on other edible *Gonimbrasia* species and their considerable contribution to the rural economies in Africa [21,22,23]. Therefore, the aim of the current study was to assess the hypothesis that the edible life stages of *G. cocaulti* and the silkworm pupae of *Bombyx mori,* and *Samia Cynthia ricini* are nutritionally dense and could serve as functional food ingredients in food fortification for fighting against possible future food insecurity in east Africa.

## 2. Materials and Methods

### 2.1. Chemicals and Reagents

All chemicals and solvents were of analytical grade unless stated otherwise. Hydrochloric acid, nitric acid, hydrogen peroxide, phosphate buffer, acetonitrile, vitamins B_2_, B_9_, B_5_, B_12_ and B_6_ standards, nicotinic acid, nicotinamide, alpha-tocopherol, beta-tocopherol retinol, potassium hydrogen carbonate, ethanol, potassium hydroxide, hexane, methanol, tetrahydrofuran, tert-butyl methyl ether, ammonium acetate, α- and γ-tocopherol standards and methyl acetate were all sourced from Sigma–Aldrich (Kobian, Kenya).

### 2.2. Sample Collection

The domesticated species of two silkworms (*S. cynthia ricini* and *B. mori*) were sampled from the sericulture rearing unit at the Animal Rearing and Containment Unit (ARCU) at icipe. At icipe, captive populations of the Eri (*S. cynthia ricini*) and domestic (*B. mori*) silkworms were reared on castor (*Ricinus communis* L.) and mulberry (*Morus alba* L.) leaves, respectively, which are the primary host plants [24,25]. From each of the two domesticated species, 2100 pupae were collected and further divided into groups of 700 each (3 groups or samples per species). The key host plant of *G. cocaulti* under field conditions, reported by Kusia et al. [19], is *Vachellia tortilis*, which is commonly called the umbrella thorn acacia or Israeli babool. A field survey on this medium to large, canopied tree yielded over 900 *G. cocaulti* caterpillar larvae in Machakos County. This collection was randomly divided into groups of 300, resulting in three groups (samples).

### 2.3. Sample Preparation

The silkworm pupae (*B. mori* and *S. cynthia ricini*) were kept separately in standard nonwoven bags (12 × 16 inches) and frozen at −21 °C in a chest freezer. While the caterpillar (*G. cocaulti)* samples collected from the field were packed in cool boxes that contained flaked ice (4–7 °C) and transported to the International Centre of Insect Physiology and Ecology (icipe) for taxonomic confirmation. The raw insect samples were then weighed into composite samples of 700 g, thawed and oven-dried (SDO-225, Wagtech International, Thatcham, UK) at 60 °C for 24 h to a moisture content of below 15%. The samples were ground in a three-speed Waring laboratory blender (Camlab, Over, UK) before being screened through a 0.1 mm stainless steel laboratory sieve. They were then vacuum-packaged in sterile Ziploc bags, labeled and stored in a deep freezer (−21 °C) until further analyses.

### 2.4. Analyses of Fatty Acids

Fatty acids in the insect meal samples were identified and quantified as their corresponding fatty acid methyl esters (FAMEs) using a 7890A gas chromatography coupled to a 5975 C mass spectrometry selective detector (Agilent Technologies, Inc., Santa Clara, CA, USA) [26]). To methylate different fatty acids, 500 µL of sodium methoxide solution (15 mg/mL) prepared in dry methanol was added to 20 mg of the sample. The mixtures were vortexed for 1 min, sonicated for 5 min and incubated at 60 °C for 1 h, thereafter quenched by adding 100 µL deionized water followed by vortexing for another 1 min. The resulting methyl esters were extracted using gas chromatography grade hexane (1000 µL; Sigma–Aldrich, St. Louis, MO, USA), centrifuged at 14,000 rpm for 5 min and the supernatant dried by passing through anhydrous sodium sulfate before analysis (1.0 µL) by gas chromatography-mass spectrometry GC-MS. The GC-MS was equipped with a (5% phenyl)-methylpolysiloxane (HP5 MS) low-bleed capillary column (30 m × 0.25 mm i.d., 0.25 µm; J&W, Folsom, CA, USA) and helium was used as carrier gas at a flow rate of 1.25 mL/min. The initial temperature of the oven was kept at 35 °C for 5 min, it was then programmed to increase to 280 °C at a rate of 10 °C/min and was held at 280 °C for 20.4 min. Ion source and quadrupole temperatures of mass selective detectors were kept at 230 °C and 180 °C, respectively and ionization energy was 70 eV. Fragment ions were analyzed over 40–550 m/z mass range in the full scan mode and the filament delay time was set at 3.3 min. The linear calibration curve of the peak area vs. concentration which gave a coefficient of determination (R2 = 0.9997) was generated by analyzing serial dilution of the authentic standard methyl octadecenoate (0.2–125 ng/µL) using chromatography coupled with mass spectrometry in full scan mode which gave the following equation, Y=5E+07x+2E+07, which was used to externally quantify different FAMEs in the samples. The FAMEs were identified by comparing mass spectral data and retention times with those of authentic standards, where available and using reference spectra published in library MS databases from National Institute of Standards and Technology (NIST) 05, 08 and 11. Absolute compound identity was assigned for matches within +/−5 of the database. Relative quantification of identified compounds was applied and individual compounds were expressed as a percentage of the total identified compounds.

### 2.5. Analyses of Minerals

The mineral contents were assessed by inductively coupled plasma emission spectrometry (ICP-AES). Each sample (5 g) was incinerated at 450 °C until its ash turned a grey to reddish-brown color. The ash was then dissolved in an acid mixture of hydrochloric and nitric acid and hydrogen peroxide (37%, 65%, 30%, respectively) as per the published protocols in [27]. The samples were analyzed for magnesium (Mg), sodium (Na), potassium (K), iron (Fe), zinc (Zn) and calcium (Ca) contents.

### 2.6. Analyses of Proximate Nutritional Parameters

The proximate components of the samples were analyzed using the protocols of AOAC [28]. The dry matter content was assessed by oven drying the sample at 105 °C till a constant weight was achieved, while moisture content was determined as the weight difference before and after oven-drying at 100 °C for 2 h. On the other hand, the total protein content of the sample was obtained by first determining the nitrogen content following the Kjeldahl method and then multiplying the nitrogen value by a conversion factor of 4.76 [29] to obtain the total protein value. Total fats were determined by diethyl ether extraction in a fat extraction unit (SER 148; VELP Scientifica, Usmate Velate, Italy) following the Randall technique. This involves mixing of the sample in hot diethyl ether, washing off the solvent after boiling and recovery by evaporation and condensation. The total ash content was determined by combustion of the sample in silica crucible at 550 °C in a muffle furnace. Finally, the acid detergent fiber (ADF) was analyzed with a VELP fiber analyzer (FIWE 6) (VELP Scientifca, Usmate Velate, Italy) using reagents described in the published protocol [30].

### 2.7. Analyses of Vitamins

The fat- and water-soluble vitamins in the various samples were analyzed following the methods described by Maiyo et al. [31] and Bhatnagar-Panwar et al. [32]. Liquid chromatography mass spectroscopy (LC-MS) coupled with a diode array detector (HPLC-30AC; Shimadzu, Tokyo, Japan) and a C18 column (100 × 3.00 mm, 2.6 µm polar; Phenomenex, Torrance, CA, USA) at 30 °C, was used and each sample and standards were analyzed for 12 min at a flow rate of 0.4 mL/min and injection volume of 10 µL. The number of water-soluble vitamins in the sample were calculated by comparing peak area of samples to peak area of the standards.

For the fat-soluble vitamins, analyses were performed using reverse-phase HPLC (Shimadzu, Tokyo, Japan) linked to a SPD-M2A detector. The injection volume of each sample was 10 µL with a total flow rate of 0.4 mL/min for 10 min. Standards for retinol and α- and γ-tocopherol were prepared at four different concentrations each and used for the calibration curve. Peaks were identified by their retention time and the absorption spectra were compared to the standardized spectra. Vitamin concentrations were calculated by comparing the peak area of samples to the peak area of the standardized spectra.

### 2.8. Statistical Data Analyses

Three replicate samples per species were analyzed for each parameter. Two statistical programs (R and PAST) were used in the statistical analyses. ANOVA and Tukey’s multiple comparisons tests (HSD) from the package “agricolae” were used to compare the assayed parameters between species. A nonmetric multidimensional scaling (NMDS) plot was completed in the PAST program (two dimensional plots, similarity measure—Bray–Curtis), to highlight the interspecies variation in free fatty acids. Box plots, bar graphs and a plot of means were completed in R v3.6.2 [33], the factoextra v1.0.6, 233 [34] and ggplot2 v3.2.1 [35] packages, to show variation of proximate components, vitamins and minerals between the species, respectively.

## 3. Results and Discussion

### 3.1. Analyses of Fatty Acids

**Figure 1:**
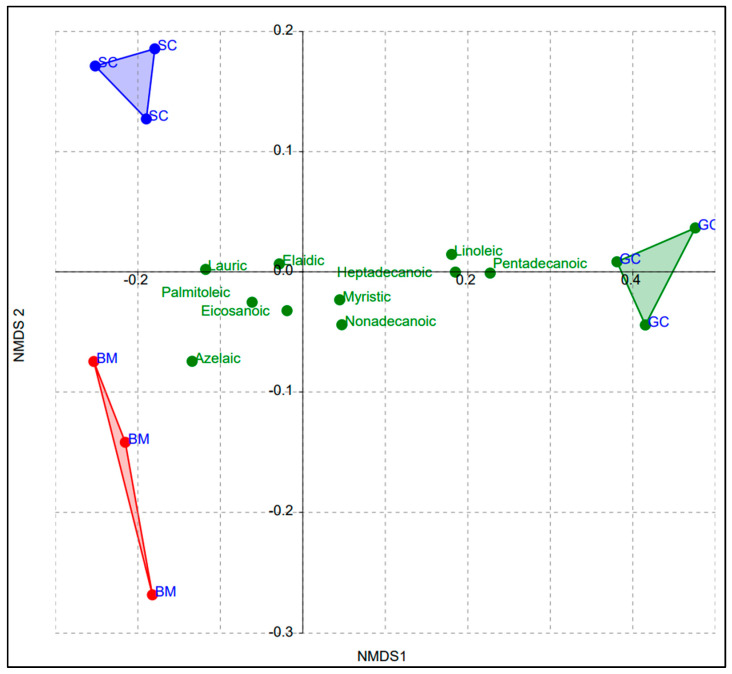
NMDS clustering of samples of edible stages of the three insects (*Gonimbrasia cocaulti*, GC; *Bombyx mori*, BM; and *Samia Cynthia ricini*, SC) based on the ten free fatty acids.

Edible insects are known as a valuable source of fatty acids, whose properties have been considered to be closer to plant than animal oils [31,36,37,38,39,40]. Ten free fatty acids were studied and were present in all three insects, though in varying concentrations. The species of the insect had a significant effect on all free fatty acids (*p* < 0.05) (Table 1 and Figure 1). In this study, lauric acid for GC was low, while its values for the silkworms were high and comparable (F = 7.146; df = 2; *p* = 0.0259). Azelaic acid was significantly higher in BM than in SC and GC, both of which had comparable values (F = 62.97; df = 2; *p* < 0.0001). Myristic and pentadecanoic acids were high in GC, while in the silkworm pupae they were low and comparable (F = 6.334; df = 2; *p* = 0.0332 and F = 391.2; df = 2; *p* < 0.0001, respectively). On the other hand, palmitoleic acid was significantly higher in BM in comparison to SC and GC (F = 23.78; df = 2; *p* = 0.00141).

The contents of both heptadecanoic (F = 298.9; df = 2; *p* < 0.0001) and linoleic (F = 109.7; df = 2; *p* < 0.0001) acids were low and comparable in the silkworms, but substantially high in GC. Elaidic acid (F = 43.08; df = 2; *p* = 0.00028) differed considerably between the three insects, while nonadecanoic (F = 5.362; df = 2; *p* = 0.0462) was low in CS but high and similar in BM and GC. Moreover, eicosanoic acid was recorded highest in BM, but low and comparable in SC and GC (F = 18.32; df = 2; *p* = 0.00279). Our findings are in agreement with fatty acid spectra reported on other edible insects [36,38,39,40]. Notwithstanding the effect of insect species, studies have also shown that the fatty acid spectra of edible insects are partly dependent on the fatty acid composition of their feed [41,42,43].

Fatty acids are essential in the body since they are the building blocks of organs, tissues and cells as well as for the synthesis of certain biologically active substances. Furthermore, they are an important source of energy, particularly, for the heart and muscular tissues through β-oxidation of fatty acids [44]. Broadly, fatty acids can be classified as saturated and unsaturated. Further, unsaturated fatty acids can either be mono- or polyunsaturated, depending on the number of double bonds in their structure [26,38]. Though the human body can synthesize de novo many of the fatty acids, some essential polyunsaturated fatty acids such as linoleic have to be delivered with diet [44].

High levels of unsaturated fatty acids in diets are recommended due to their positive multidirectional effects in the body [44,45]. Whereas, high levels of saturated fatty acids in diets are not favorable because they raise the level of low-density lipoproteins [46,47]. Therefore, due to the high levels of unsaturated fatty acids (palmitoleic, linoleic and elaidic acids) in the studied insects, their consumption either as food or feed, may counter the possible negative effects of the saturated fatty acids that were also present. For example, cumulatively GC had a significantly higher amount of unsaturated fatty acids (fivefold) compared to saturated fatty acids (Table 1).

The samples from the three insects separated into distinct convex hulls, with GC samples being highly influenced by five free fatty acids: nonadecanoic, myristic, linoleic (polyunsaturated), heptadecanoic and pentadecanoic (Figure 1). In contrast, BM was influenced by eicosanoic, azelaic and palmitoleic, while SC was affected by lauric and elaidic. Broadly, the silkworm pupae were separated to the negative side of NMDS 1, while the GC caterpillars were separated to the positive side. This clearly signifies that the insect species and its developmental stage and age had a direct influence on the type and amount of free fatty acids that were present in the three insects. This is in agreement with the findings of previous studies, which have shown that the nutritional components of edible insects vary based on species and age among other factors [26,41,42,43,48].

### 3.2. Analyses of Minerals

**Figure 2:**
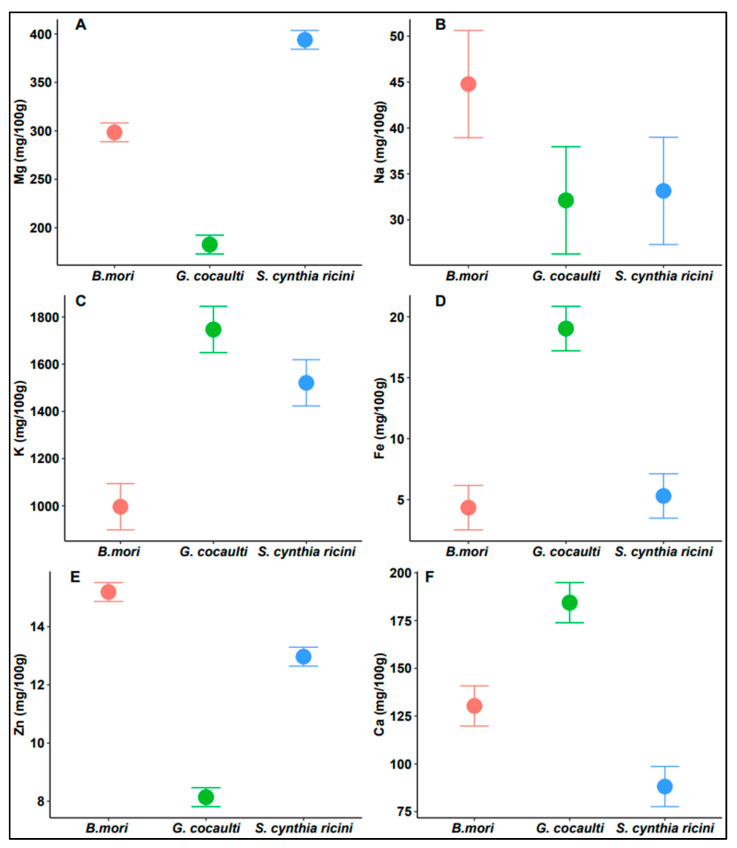
Mean plots showing variation in major minerals across the three edible insects. Where (**A**) is magnesium (Mg), (**B**) is sodium (Na), (**C**) is potassium (K), (**D**) is iron (Fe), (**E**) is zinc (Zn) and (**F**) is calcium (Ca).

The three insects exhibited different accumulation patterns of minerals (Figure 2), which is primarily attributable to seasonal, geographic and ecological factors as the minerals are not synthesized in the insect body but rather obtained from the dietary sources [49,50,51].

Potassium (F = 165.7; df = 2; *p* < 0.0001), iron (F = 218.2; df = 2; *p* < 0.0001) and calcium (F = 225.8; df = 2; *p* < 0.0001) were high in GC (Figure 2C,D,F), whereas sodium (F = 15.54; df = 2; *p* = 0.00423) and zinc (F = 1320; df = 2; *p* < 0.0001) were found to be high in BM (Figure 2B,E). *Samia cynthia ricini* recorded higher amounts of magnesium (F = 1280; df = 2; *p* < 0.0001) compared to the other two insects (Figure 2A). These findings reveal that these insects are rich sources of essential minerals, however, GC stands out as a better source, which may be because it has a wide range of food sources available for its ingestion in the wild. The mineral levels detected in this study were consistent with the results of other studies in the literature on edible insects [22,49,50,51,52,53]. Moreover, the obtained mineral levels were comparable or even superior to most products of animal origin [39,54].

Among the studied mineral elements, Ca, Mg and Fe are key markers of the nutritional value of a given food in the aspect of its mineral composition, since they are the most frequently reported mineral deficiencies [53]. For instance, Ca plays vital roles in many physiological functions such as blood clotting, bone and tooth formation, enzymatic activity, metabolic reactions, construction of proteins, muscle contraction and membrane permeability [54,55,56]. The main sources of Ca are dairy products; however, they are beyond the reach of the many poor populations in developing countries. Since the Ca content of the studied insects, particularly GC, compared favorably with conventional sources, they could be considered as possible sources for enriching diets. It is also significant to note that Ca from insect can be an appropriate substitute for people with lactose intolerance and allergies to soy [57]. In poultry feeds, Ca is crucial to bone and egg formation as it contributes up to 90% of their mineral matrices [58].

Iron deficiency is common among infants, young children, young women and the elderly [54]. For example, earlier reports showed that in UK, iron deficiency in young women (11 to 18 years) was 21%, while in USA 9–11% of nonpregnant women (16–49 years) were anemic and in 2–5% of them, the anemia was related to iron deficiency [59]. In some African countries (Kenya, Uganda, Burkina Faso and Gambia), the data on prevalence of anemia ranged between 49.7% and 87.0% [60]. The high amounts of Fe recorded in this study, especially in GC, whose Fe content (>15 mg/100 g) was superior to conventional meat sources and comparable if not higher than those of other edible insects [51,53,54], is indicative that consummation of these insects will help alleviate such deficiencies.

The high Mg levels in the three insects is also beneficial for calcium metabolism and proper operation of the heart, among many other roles, if consumed as feed or food [61]. Equally, Na which is important in controlling blood pressure and minimizing the risk of coronary disease, was present within the required range [62]. Zinc a key component of many enzymes and essential in the metabolism of major nutrients (proteins, fats and carbohydrates), was present in comparable levels with other sources of animal origin [53,63]. Moreover, Zn plays a crucial role in polynucleotide transcription, thus critical in the process of genetic expression [54]. Potassium is similarly critical in reducing high inflow of blood in the blood vessels, thus preventing cardiovascular related diseases [63].

### 3.3. Analyses of Proximate Nutritional Parameters

**Figure 3:**
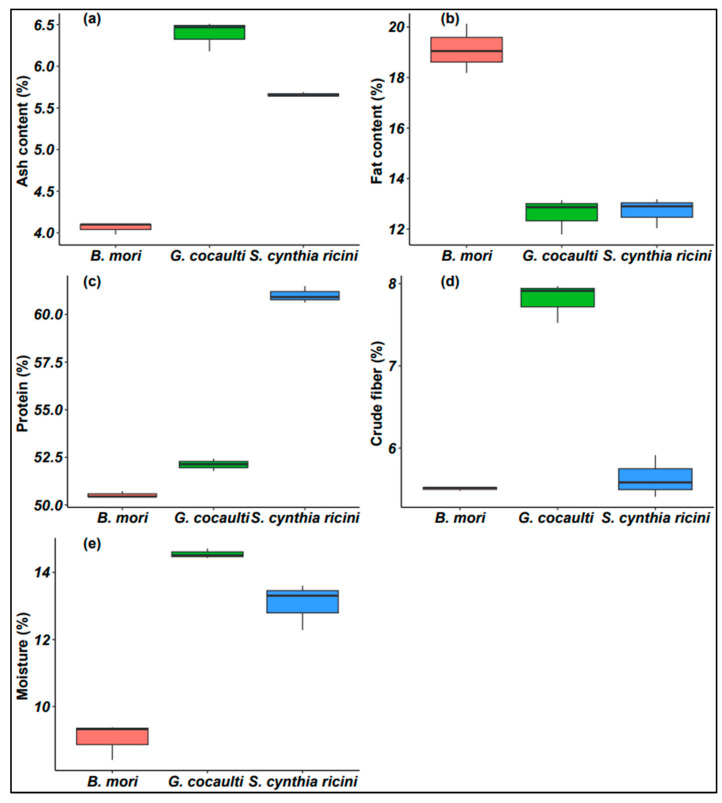
Box plots for the proximate analyses of the edible stages of the three insects. Where (**a**) is ash content, (**b**) is fat content, (**c**) is protein, (**d**) is crude fiber and (**e**) is moisture. All are represented in percentage (%) dry matter basis.

The findings of proximate nutrients analyzed separately on a dry matter basis are given in Figure 3. There was an effect of insect species in all the proximate nutritional parameters. The ash content (F = 336.2; df = 2; *p* < 0.0001) and crude fiber (F = 118.4; df = 2; *p* < 0.0001) were both highest in GC and lowest in BM, nevertheless, the crude fiber values in the silkworms were comparable. The silkworm BM possessed the highest values in both the dry matter (F = 90.98; df = 2; *p* < 0.0001) and fat content (F = 68.83; df = 2; *p* < 0.0001), while GC had the lowest values, even though the fat content values in GC and SC did not differ significantly. The protein content (F = 874.0; df = 2; *p* < 0.0001) was noticeably higher in SC compared to the other two insects. However, it is important to note that all three insects had extremely high-protein content (>50%). The highest moisture content (F = 90.98; df = 2; *p* < 0.0001) was recorded in GC and lowest in BM.

Protein content was dominant in all three insects, followed by total fat. These findings are comparable to those reported by Arasakumar et al. [64] on the protein content of BM and SC, though they reported higher fat content than what we found. These variations could be due to the differences in habitat, climate, sex, processing method and genetic variability [26,42,43]. Studies have shown that the protein content of insects ranges between 25 and 75% [65,66,67,68], which corroborate results of this study. However, it is important to note that the protein values in the current study were significantly higher when compared to some animal and plant protein sources such as milk, beef, roasted goat meat, broiler, pork, eggs, dried whey protein concentrate and soybeans, in which protein constitute between 24 and 45% [39,69,70]. Furthermore, in relation to the high digestibility and desirable amino acid profile (building blocks of protein), insect proteins are rated highly [12]. A higher protein digestibility signifies a higher availability of amino acids, particularly, indispensable (essential) amino acids, which cannot be synthesized by most animal species, hence must be delivered through diet [71]. Therefore, the edible stage of GC is a good and cheap source of protein, as are the silkworms and other edible insects.

The fat content of insects has been reported to vary between 10 and 70% on a dry matter basis, which is within the range reported in the current study [53,64,66]. However, the obtained fat content values for SC were lower than those reported in the literature [72,73,74]. Fat is made of fatty acids that can either be saturated, monounsaturated, or polyunsaturated and they play vital physiological roles, as earlier discussed, for free fatty acids. Although insects are largely viewed as an alternative source of protein, many of them are rich in valuable oils, which if used together with other foods could increase their nutritive value [74].

The ash content levels agree with those reported by other authors investigating various edible insects [48,53,68,70,72]. There is a consensus among researchers that ash content of a given sample correlates with its specific mineral elements, which agrees with our findings. For instance, GC had a higher ash content than the silkworms and was equally found to be high in three mineral elements (K, Ca and Fe) (Figure 2). The considerable levels of ash in the studied insects are indicative that they may be a good source of minerals and therefore their inclusion in processed food products has the potential to enrich their mineral content [54]. The crude fiber content was consistent with previous outcomes, in which most edible insect species had a crude fiber content within the desired range [50,52,75,76]. Insect fiber is mainly made up of protein and considerable amounts of chitin. The latter is indigestible despite the presence of the enzyme chitinase in human gastric juice, particularly, in people from nontropical countries where this enzyme is inactive [77]. However, people from tropical countries with a long history of eating insects have active chitinase and thus can digest insect fiber [78]. Chitin digestion and removal increases insect protein digestibility [79]. The moisture content of the powders was less than 15%, which is vital as it reduces the threat of microbial contamination, thus increasing the preservation period. Previous studies have observed a similar trend in moisture content in various edible insect species [50,68]. In contrast to the current findings, a higher moisture content has been reported in other edible insects, which could be due to factors such as geographical location and habitat [54,62].

**Figure 4:**
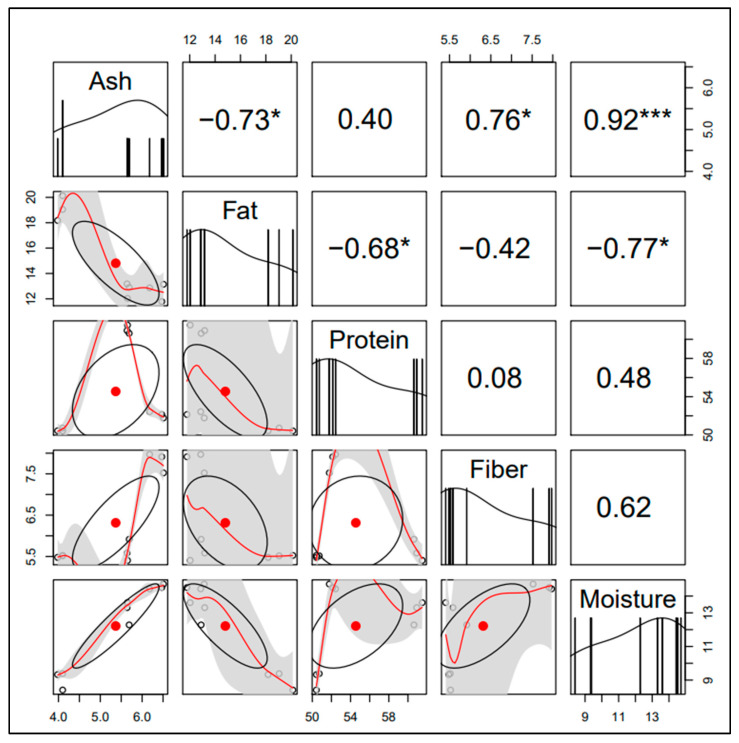
Spearman rank (r_s_) correlations for proximate nutritional components. Where a star or stars shows those with statistical significance; * *p* < 0.05, *** *p* < 0.001.

There were high-positive associations between the ash content with fiber and moisture contents of 0.76 (*p* < 0.05) and 0.92 (*p* < 0.001), respectively (Figure 4). However, high-negative associations were recorded between ash content and fat (−0.73; *p* < 0.05. Additionally, there were high-negative associations observed between fat and protein (−0.68; *p* < 0.05) and with moisture content (−0.77; *p* < 0.05). Fiber had a positive association with moisture (0.62), though this association was not significant (*p* = 0.077). These associations show that some of the proximate components are related and that an increase in one may lead to a decrease in the other and vice versa. For instance, if there is more fat content then one should expect less protein and ash in the studied insects, since they are inversely associated.

### 3.4. Analyses of Vitamins

**Figure 5:**
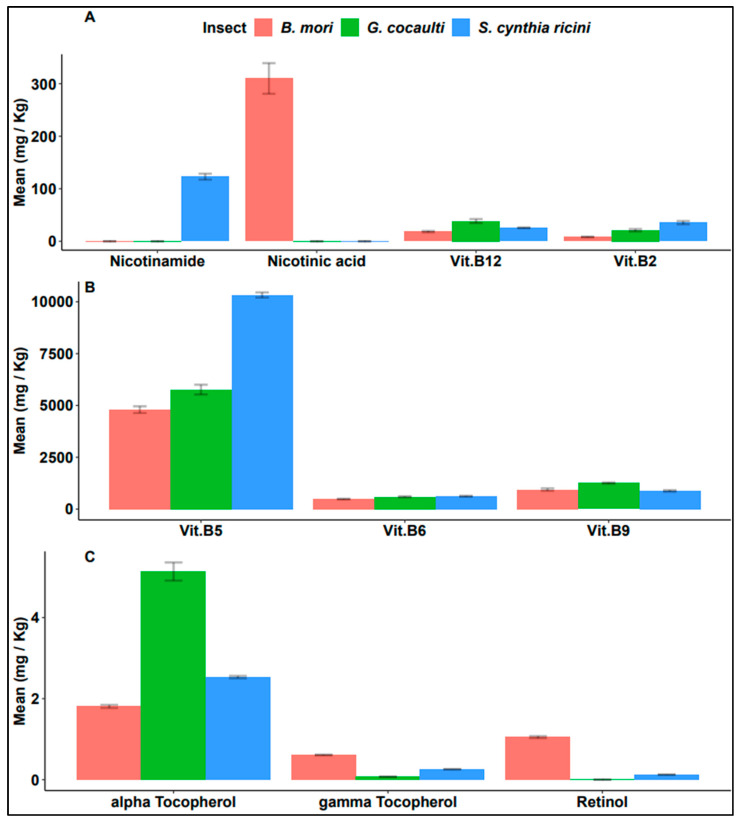
Variation in the concentration of different vitamins; (**A**,**B**) water-soluble vitamins and (**C**) lipid-soluble vitamins, across the three African insects’ edible stages.

Water-soluble vitamins were present in all samples except nicotinamide which was absent in BM and GC and nicotinic acid which was absent in GC and SC samples (Figure 5A,B). On the other hand, fat-soluble vitamins were equally present in all samples except retinol that was absent in GC samples (Figure 5C).

Vitamins B_2_ and B_5_ were both highest in SC and lowest in BM samples and were significantly different in concentration across the three insects (F = 111.9; df = 2; *p* < 0.0001 and F = 809.6; df = 2; *p* < 0.0001, respectively). Further, the insect species had a significant influence on vitamins B_9_ (F = 64.73; df = 2; *p* < 0.0001) and B_12_ (F = 58.21; df = 2; *p* = 0.00012) both of which were highest in GC but were lowest in the silkworms. Vitamin B_6_ (F = 34.72; df = 2; *p* = 0.0005) was recorded lowest in BM, while its values for GC and SC samples were high and comparable. Retinol (F = 3925; df = 1; *p* < 0.0001) was only present in the silkworms, though it was higher in BM than SC. Alpha-tocopherol was higher in GC compared to the two silkworms, while gamma-tocopherol was highest in BM and lowest in GC. The observed differences in the vitamin contents between the studied insects could be due to variation in the diet eaten by the insects [80]. Our results agreed with the findings reported by other authors investigating vitamins in various edible insects [26,62].

Any feed or food needs to contain sufficient quantities of vitamins, as they facilitate the development of proper and healthy body functions. Moreover, vitamins contribute to the development of the immune system, while also helping in the digestion of other nutrients [81,82]. The fat-soluble vitamins (α and γ tocopherols) are good natural antioxidants that impede lipid oxidation in biological systems by stabilizing hydroperoxyl and in mopping out other free radicals [83,84,85]. As vitamins cannot be synthesized de novo by animals, they must be supplied constantly by food, thus incorporation of the studied insects in diets would likely serve as an alternative source of vitamins.

In addition to macro- and micronutrients, edible insects are rich in natural bioactive compounds, which includes peptides and phytochemicals [18,86]. These compounds have unique and yet vital properties such as hydrophilicity, permeability and multifactorial interaction with the biological molecules, all of which are essential in disease prevention [84]. Previous studies have shown that synthetic bioactive compounds may impede other nutrients’ biological functions in vivo, therefore, natural compounds are often regarded as being safer [86]. For instance, a recently published in vitro study revealed two peptides extracted from silkworm pupae powder, which has strong antioxidant activity in HepG2 cells [87]. Furthermore, most compounds in silkworm pupae have been reported to have various pharmacological functions, which includes antibacterial, antitumor, antioxidant, lipid and blood-sugar-regulation, antiapoptotic, hypotensive, improving liver function, antifatigue, antiaging, antigenotoxic, alcohol detoxification and immunomodulatory effects [86,88,89]. Therefore, it could be possible to develop functional foods or medicines from silkworm pupae extracts, to help prevent diseases (diabetes, obesity and other lifestyle related disorders) caused by oxidative stress [88,89]. Unfortunately, such extensive studies described above on silkworm pupae, have never been researched for *Gonimbrasia* spp., which opens new opportunities for future efforts.

It is also important to note that most of the aforementioned silkworm pupal bioactivities have only been tested in vitro, hence in vivo assays either in animal models or human are needed. Despite the huge potential of edible insects as food and feed or medicine, their safety needs to be evaluated, both for toxicological safety and for allergic reactions. For instance, though insect oils are high in unsaturated fatty acids, they are not recommended for direct human consumption due to the presence of triacylglycerols, which if consumed may increase the risk of heart related diseases [90]. In addition, allergenicity has been described in some edible insects such as peptides such as 27-kDa glycoprotein, chitinase precursor and profilin in silkworms [91,92]. However, processing through heat could cause chemical modifications to the allergens, resulting in the loss or reduction in allergenicity [93]. Another important aspect that needs to be considered is the development of laws and regulations on consumption of edible insects, especially in Africa. In Europe, the new EU food legislation allows for the legalization of edible insect consumption [94]. Such regulations are needed to govern the production and consumption of insect and as a form of protection for consumers.

## 4. Conclusions

The overall nutrient profile of the edible stage of GC was comparable to that of the silkworm pupae, which were comparable to, or surpassed, those of the conventional animal and plant-based products in some cases. The high content of mono- and polyunsaturated fatty acids, vitamins, minerals and protein in the targeted edible stage of GC and silkworm pupae showed that they could be explored as a food additive for tackling nutritional deficiencies. However, further studies on protein digestibility, amino acid profile, antinutrients and antioxidants would be necessary. With the ongoing challenges of an expanding global population, climate change, scarcity of land and water and the ever increasing undernourished populations in developing countries, novel food production strategies are required. The rearing of edible insects for food and feed has the potential to bridge these gaps in an environmentally sustainable manner.

## Figures and Tables

**Table 1:** Concentration of free fatty acids at the edible stages of the three African insects (mean of % peak area ± standard deviation).

Free Fatty Acids	Insects					
	*B. mori*	*S. cynthia ricini*	*G. cocaulti*	F-Value	df	*p*-Value
Lauric	0.23 ± 0.07 ^b^	0.23 ± 0.06 ^b^	0.08 ± 0.04 ^a^	7.146	2	0.0259
Azelaic	2.82 ± 0.41 ^b^	0.96 ± 0.07 ^a^	0.53 ± 0.20 ^a^	62.97	2	<0.0001
Myristic	1.80 ± 0.48 ^ab^	1.24 ± 0.10 ^a^	2.04 ± 0.02 ^b^	6.334	2	0.0332
Pentadecanoic	0.33 ± 0.09 ^a^	0.35 ± 0.02 ^a^	1.52 ± 0.03 ^b^	391.2	2	<0.0001
Palmitoleic	9.47 ± 1.14 ^b^	6.57 ± 0.64 ^a^	4.97 ± 0.51 ^a^	23.78	2	0.0014
Heptadecanoic	1.89 ± 0.11 ^a^	1.85 ± 0.12 ^a^	6.18 ± 0.40 ^b^	298.9	2	<0.0001
Linoleic	4.52 ± 0.35 ^a^	6.99 ± 1.16 ^a^	18.18 ± 1.69 ^b^	109.7	2	<0.0001
Elaidic	68.88 ± 3.10 ^b^	76.08 ± 1.42 ^c^	59.03 ± 1.92 ^a^	43.08	2	0.0003
Nonadecanoic	1.69 ± 0.65 ^a^	0.81 ± 0.07 ^b^	1.69 ± 0.07 ^a^	5.362	2	0.0462
Eicosanoic	8.36 ± 1.15 ^b^	4.92 ± 0.32 ^a^	5.78 ± 0.36 ^a^	18.32	2	0.0028

Where, df = degree of freedom and different letters in the same row shows statistical significance between the values.

## Data Availability

Data used in this study is contained within the article and is freely available upon request from the corresponding author.
